# Characterizing Retinal Ganglion Cell Responses to Electrical Stimulation Using Generalized Linear Models

**DOI:** 10.3389/fnins.2020.00378

**Published:** 2020-05-12

**Authors:** Sudarshan Sekhar, Poornima Ramesh, Giacomo Bassetto, Eberhart Zrenner, Jakob H. Macke, Daniel L. Rathbun

**Affiliations:** ^1^Institute for Ophthalmic Research, Eberhard Karls University Tübingen, Tübingen, Germany; ^2^Graduate Training Center of Neuroscience, International Max Planck Research School, Tübingen, Germany; ^3^Systems Neuroscience Center, School of Medicine, University of Pittsburgh, Pittsburgh, PA, United States; ^4^Department of Bioengineering, University of Pittsburgh, Pittsburgh, PA, United States; ^5^Center for the Neural Basis of Cognition, University of Pittsburgh and Carnegie Mellon University, Pittsburgh, PA, United States; ^6^Computational Neuroengineering, Department for Electrical and Computer Engineering, Technische Universität München, Munich, Germany; ^7^Neural System Analysis, Research Center Caesar, Bonn, Germany; ^8^Werner Reichardt Centre for Integrative Neuroscience, Tübingen, Germany; ^9^Bernstein Center for Computational Neuroscience Tübingen, Tübingen, Germany; ^10^Department of Ophthalmology, Henry Ford Health System, Detroit, MI, United States

**Keywords:** retina, prosthetics, white-noise stimulation, generalized linear models, SNR, nested models

## Abstract

The ability to preferentially stimulate different retinal pathways is an important area of research for improving visual prosthetics. Recent work has shown that different classes of retinal ganglion cells (RGCs) have distinct linear electrical input filters for low-amplitude white noise stimulation. The aim of this study is to provide a statistical framework for characterizing how RGCs respond to white-noise electrical stimulation. We used a nested family of Generalized Linear Models (GLMs) to partition neural responses into different components—progressively adding covariates to the GLM which captured non-stationarity in neural activity, a linear dependence on the stimulus, and any remaining non-linear interactions. We found that each of these components resulted in increased model performance, but that even the non-linear model left a substantial fraction of neural variability unexplained. The broad goal of this paper is to provide a much-needed theoretical framework to objectively quantify stimulus paradigms in terms of the types of neural responses that they elicit (linear vs. non-linear vs. stimulus-independent variability). In turn, this aids the prosthetic community in the search for optimal stimulus parameters that avoid indiscriminate retinal activation and adaptation caused by excessively large stimulus pulses, and avoid low fidelity responses (low signal-to-noise ratio) caused by excessively weak stimulus pulses.

## 1. Introduction

Age-related macular degeneration (AMD) and retinitis pigmentosa (RP) are two common retinal degenerative diseases that cause profound vision loss (Lorach et al., 2015). Both these diseases lead to blindness through photoreceptor death. However, studies have shown that the retinal circuitry in the inner plexiform layer (IPL) remains relatively intact, despite undergoing significant rewiring (Jones et al., 2003; Jones and Marc, 2005; Gargini et al., 2007). Though there is not yet a cure for these diseases, multiple treatment options are currently being investigated. One such approach involves the use of electrode arrays implanted in the eye (also known as retinal implants) that electrically stimulate the diseased retina. Retinal implants have been able to restore some degree of visual perception back to patients (Zrenner et al., 2011; Humayun et al., 2012; Stingl et al., 2015). These prosthetic devices can either directly target the retinal ganglion cells (RGCs) or stimulate the retinal network in order to use the remnant visual processing present in the IPL.

At present, retinal implants (regardless of the intended site of stimulation), use a train of constant amplitude current or voltage pulses, with individual pulses designed to elicit retinal activity. RGCs were recently shown to be able to integrate a stream of smaller subthreshold pulses via the retinal network, in order to generate spiking responses (Sekhar et al., 2016). Moreover, by combining spike-triggered averaging (STA) with this subthreshold stimulus paradigm, different RGC classes (ON- and OFF-cells) were found to have distinct electrical input filters (Sekhar et al., 2017). While STA-like waveforms could be used to selectively stimulate different RGC classes, classical spike-triggered techniques provide only a coarse characterization of neural responses to stimuli, for the following reasons: First, major underlying assumptions of STA analyses are that the stimuli have a white-noise power spectrum (Chichilnisky, 2001) and that the interaction between applied stimuli and evoked responses is linear. Such a model can not capture non-linear interactions that may exist between the stimulus and the neural response (Gollisch and Meister, 2010; da Silveira and Roska, 2011), and might be biased if the stimuli are not white noise. Second, fluctuations in firing rates of cells across trials constitute a complicating factor. Long duration, high frequency electrical stimulation can alter the responsiveness of the retina over the course of a recording. Thus, models fit to the first half of a recording might perform poorly at predicting the responses during the second half, and vice versa. Third, statistical estimators based on spike-triggered averaging can be less data-efficient compared to likelihood-based approaches (Paninski, 2004). Fourth, STA-analyses do not allow one to incorporate prior knowledge or assumptions about likely filter-shapes (e.g., their temporal smoothness) into the estimation procedure, implying that larger data-set sizes (i.e., longer recording times) are required for characterization.

Therefore, while STAs are a useful step in describing a cell's stimulus-response function, model-based approaches (e.g., based on generalized linear models) confer additional advantages. In particular, they can be used to quantify how well variations in firing rate can be captured by a linear model, and how much can be attributed to non-stationarity in RGC firing rates. This non-stationarity or trial to trial variability can arise from multiple sources, such as adaptation to stimulation, or the fact that subthreshold stimuli drive responses with much lower fidelity.

In this paper we address these issues by investigating how reliably subthreshold stimuli are able to drive RGC responses, and how different sources of RGC response variability (linearity, non-linearity and non-stationarity) depend on RGC type (ON, OFF, ON-OFF). To this end, we model the RGC responses to electrical stimuli using generalized linear models (GLMs) (Nelder and Wedderburn, 1972; Chornoboy et al., 1988; Paninski, 2004; Pillow et al., 2005; Truccolo et al., 2005). GLMs model the neural responses as follows—first, predicted firing rates are calculated by linearly combining the input stimulus values and transforming them via a non-linear function; next, the firing rates are used as parameters to sample spike counts from a Poisson or Bernoulli distribution. These models have been extensively used to describe the dependence of firing rates of retinal ganglion cells on visual stimuli (Paninski, 2004; Pillow et al., 2008; Gerwinn et al., 2010; Park and Pillow, 2011). The GLM modeling framework makes it possible to impose constraints on the parameters, yielding more useful receptive field estimates compared to the classical spike-triggered approach, and also requires less data to converge (Paninski, 2003; Gerwinn et al., 2010; Park and Pillow, 2011). We set up a hierarchy of increasingly complex GLMs to capture different factors which might be predictive of neural firing rates, namely non-stationarity across trials, linear stimulus dependence and any residual non-linear dependence on the input stimulus. To tackle non-stationarity—the issue of fluctuating firing rates across trials—we set up a GLM with one parameter for every trial. This model approximately captures the average firing rate within each trial. We then added Linear-Non-linear Poisson filter parameters to the non-stationarity (NS) model, which captures a linear response of the neuron to the external stimuli. Finally, we added one parameter for every time bin to the previous model, which makes it possible to capture any stimulus-dependent response of the neuron which is consistent across multiple trials.

**Figure 1:**
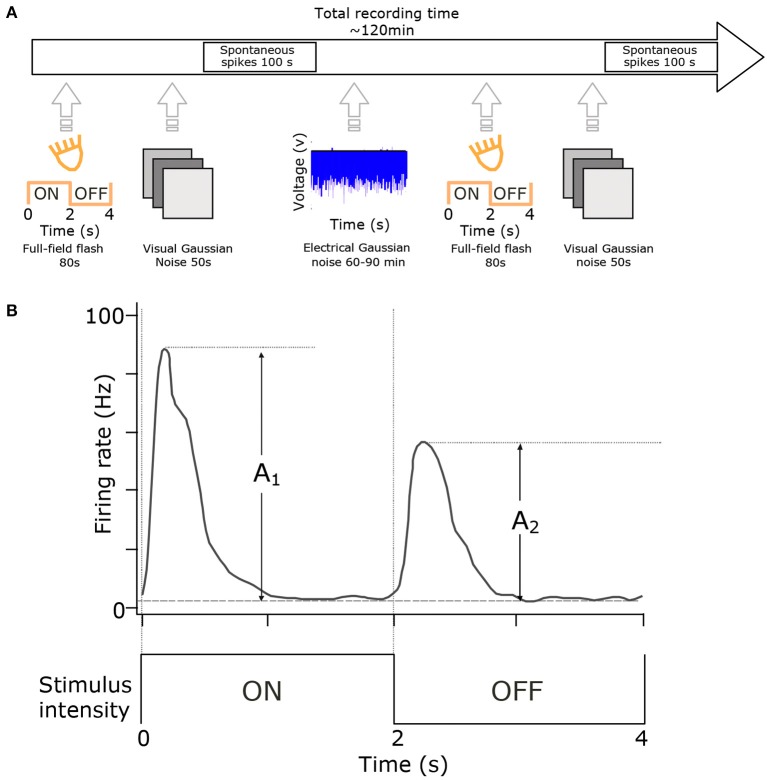
Experimental setup and cell-type classification. **(A)** RGCs were classified using full-field flash (20 repetitions of 2 s ON and 2 s OFF) and visual full-field Gaussian noise (50 s at 10 Hz). These stimuli were presented at the start and end of each experiment. The primary stimulus was at least one hour of electrical noise presented in 100 s blocks. **(B)** Cell classification. Histograms of cell responses (spike-times) during flash stimuli were quantified using the Carcieri method (Carcieri et al., 2003). Figure adapted with permission from Sekhar et al. (2017).

**Figure 2:**
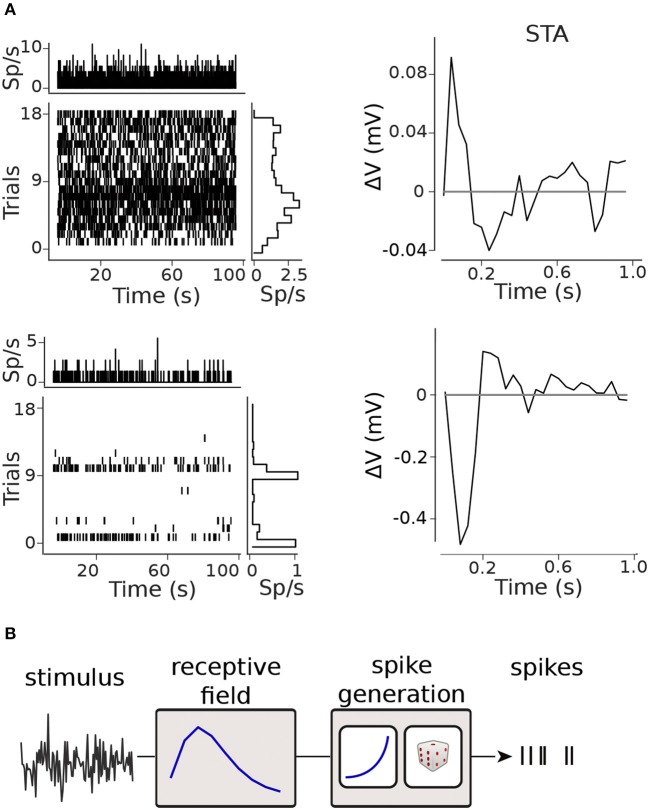
Modelling framework. **(A)** Raster plots and spike-triggered averages (STAs) for two retinal ganglion cells (RGCs): the raster plots shows moderate (top) and marked (bottom) inter- and intra-trial variability in the firing rate; clear STAs were obtained in both cases. **(B)** Generalized Linear Model (GLM) framework.

We fit these models to data recorded from mouse retinal ganglion cells stimulated with sub-threshold electrical white noise (see Figure 1). We found that our models were able to capture between 5 and 15% of the total variability in spiking responses even for neurons with very low or highly varying firing rates (see Figure 2A). However, a large fraction of the spiking variability was left unexplained even by the most complex model, indicating that most of the variability in neural responses is not explained by the stimulus. In summary, this study represents the first attempt to systematically characterize the non-stationarity, linearity and non-linearity of different RGC types under high-frequency subthreshold white noise electrical stimulation.

## 2. Methods

### 2.1. Experimental Design

The experimental methods used in this paper are identical to a previous study (Sekhar et al., 2017), and are restated here.

#### 2.1.1. Electrical Stimulus

We recorded retinal ganglion cells responses to white-noise electrical stimulation during 36 trials that were 100 s long, for a total recording time of 1 hour. This duration was previously determined to represent an acceptable compromise in which enough data is acquired to yield statistically significant electrical STAs, while at the same time, allowing enough subsequent recording time for flash and visual noise stimuli, which are used to estimate visual cell-type. Electrical white noise (−800/280*mV*, mean/sd) was delivered with a frequency of 25 Hz (pulse width 1 ms). A fixed realization of white noise was used for half of the trials, interleaved with trials where a new, previously unseen white noise sequence was delivered on each trial. The impedances of the electrodes in saline tested between experiments were ~200–250 kΩ at 1 kHz using a NanoZ impedance tester (Plexon Inc., TX, USA).

#### 2.1.2. Visual Stimulation

Visual stimulation was presented before and after electrical stimulation for cell-classification purposes (ON, OFF, ON-OFF). Flash stimulus blocks consisted of cycling 2 s ON (40 klx) and 2 s OFF (20lx) full-field luminance (mean luminance 20 klx, 99.9% Michelson contrast). Visual stimuli were presented with a linearized, commercially available DLP-based projector (K10; Acer Inc., San Jose, California, USA). Other than during visual stimulation, a shutter was placed in front of the projector.

#### 2.1.3. Animals

The data in this paper consists of 58 RGCs obtained from 16 retinal pieces using 15 C57BL/6J mice. The age of the mice are 2 × *P*32, 2 × *P*39, 2 × *P*46, 1 × *P*49, 1 × *P*51, 1 × *P*53, 1 × *P*56, 2 × *P*58, 2 × *P*59, 1 × *P*64. All experimental procedures have the approval of the state authorities (*Regierungspraesidium*, Tübingen) and were conducted under the supervision of the Tübingen University facility for animal welfare (*Einrichtung für Tierschutz, Tierärztlichen Dienst und Labortierkunde*) and the NIH Guide for the Care and Use of Laboratory Animals.

#### 2.1.4. Data Collection

The mice were anesthetized using CO_2_ inhalation and subsequently sacrificed by cervical dislocation. The eyes were then removed and subsequently dissected. The dissection process (performed under dim light conditions) consisted of removing the cornea, ora serrata, lens and vitreous body. Following this, the optic nerve was cut at the base of the retina and the retina was detached from the pigment epithelium. All traces of vitreous material were then removed from the inner surface of the retina. The retinas were then perfused with carbogenated artificial cerebrospinal fluid (ACSF) which was regulated at 33°C (using a heating plate and heated perfusion cannula) and at a pH of 7.4. Retinal pieces were mounted ganglion cell side down on a standard 60-channel microelectrode array (MEA, 60MEA200/30iR-ITO, Multi Channel Systems, Reutlingen, Germany), and were constantly perfused with ACSF. A single electrode was used for electrical stimulation and analysis was restricted to the 7-8 electrodes immediately surrounding the stimulating electrode (inter-electrode distance 200 or 283 μm), in order to ensure that all cells analyzed were exposed to stimuli of comparable strength. Voltage traces were sampled with MultiChannel Systems hardware (MCS, Reutlingen, Germany) at a rate of 50kHz/channel, using a filter bandwidth of 1–3 kHz and a gain of 1,100.

#### 2.1.5. Pre-processing and Inclusion Criteria

Raw data was high-pass filtered in order to extract putative action potential events (spikes). Next, these putative spikes underwent automated and manual spike sorting in order to reduce Type I and Type II errors in assigning waveforms to different sources. Finally, a cell validation score was used to determine whether the various clusters could be deemed as well-isolated spike trains or not. The cell validation score was calculated based on: (1) the presence of a clear lock-out period in the ISI histogram and autocorrelogram; (2) the absence of a peak in the cross-correlogram between different cells, which would indicate that a single cell had been wrongly split into two or more units; (3) good separation in principal component space of a biphasic waveform, whose shape is typical of extracellularly recorded action potentials; (4) stability of the waveform shape and firing rate over the entire experiment. To be included in this study, a cluster (putative spike train) had to score 2.5 or more in the cell validation step (on a scale from 1 to 5) and had to have a statistically significant STA. A previous study (Sekhar et al., 2017) describes in detail the method for determining if the STA of a well-isolated cluster is statistically significant. Offline Sorter (Plexon Inc, TX, USA) was used to filter and spike sort the data. Time stamps of these sorted spikes were collected with NeuroExplorer (Plexon Inc, TX, USA) and exported to MATLAB.

For all included cells, we discretized the spike trains into bins of size Δ*t* = 40*ms*. The bin onset was aligned to the electrical stimulus time stamp. The spikes falling within the first 10ms after stimulus delivery were discarded to minimize those spikes that might have been elicited due to direct activation of the RGCs. The remaining spikes were counted, and their number assigned to the corresponding bin value. The final data set consisted of 36 trials containing *N* = 2, 500 time bins for each cell.

### 2.2. Data Analysis

#### 2.2.1. Generalized Linear Model

To model neural spiking activity, we discretize the time-axis into bins of size Δ*t* = 40*ms*, and refer to $y_{t}^{i}\in \left\{0,1,2,\ldots \right\}$ as the number of spikes on trial *i* in the bin indexed by *t*, i.e., in the time-window [Δ*t*·(*t*−1), Δ*t*·*t*]. We modeled the spike count in bin *t* of the *i*-th trial as $y_{t}^{i}$ ~ Poisson(Δ*t*$\lambda_{t}^{i}$), where $\lambda_{t}^{i}$ is the instantaneous firing rate of the neuron. Following the Generalized-Linear Model (GLM) formalism (Nelder and Wedderburn, 1972; Brown et al., 2003; Truccolo et al., 2005), depicted in Figure 2B, we modeled $\lambda_{t}^{i}=f(z_{t}^{i})$), where $f(z_{t}^{i})$ is a non-linear, monotonically increasing function of an instantaneous activation variable $z_{t}^{i}$. The activation is calculated by a linear combination of covariates ${\text{x}}_{t}^{i}$ (stimulus) and coefficients **θ**,

(1) $$\begin{array}{l} z_{t}^{i}={{\text{x}}_{t}^{i}}^{⊤}\theta . \end{array}$$

We used the canonical inverse link function for a Poisson GLM, i.e., *f*(*z*) = exp(*z*).

We constructed a series of nested models which capture increasingly complex properties of the neural response, and quantified their performance in capturing neural responses to electrical stimulation:

#### 2.2.2. Baseline Model (BS)

The baseline model constitutes a reference against which the performance of all other models was compared. In the baseline model, the firing rate is a constant, which is simply the average firing rate of the neuron, i.e.,

(2) $$\begin{array}{l} \lambda_{t}^{i}=e^{b_{o}}=\frac{n_{sp}}{TK}, \end{array}$$

where *b*_*o*_ is the log firing rate, *n*_*sp*_ is the total spike count of the neuron; *T* is the number of time-bins and *K* is the number of trials.

#### 2.2.3. Non-stationarity (NS)

**Figure 3:**
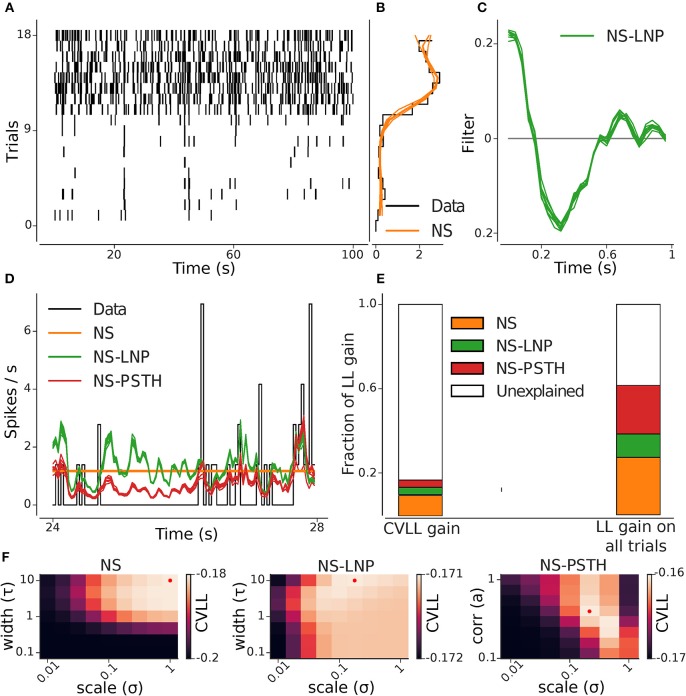
GLM hierarchy fit on example cell. **(A)** Raster plot of the repeating stimulus trials: this cell shows a high degree of non-stationary. **(B)** Cross-validated (orange) and empirical (black) per-trial firing rate. **(C)** Stimulus filter estimated from the NS-LNP model. **(D)** Empirical (black) and predicted average firing rate across trials: NS (orange), NS-LNP (green), and NS-PSTH(red). **(E)** Average gain in log likelihood for all the models from cross validation (left) and on training data (right) as a fraction of the total explainable log likelihood gain (see section 2.2). **(F)** Cross-validated log-likelihood landscapes for a grid of hyper-parameters: the red dots mark the best hyper-parameter set for a given model.

To capture slow fluctuations in neural activity (Tomko and Crapper, 1974; Czanner et al., 2008; Park et al., 2015) (Figure 2A) we augmented the baseline model with separate per-trial offset ϕ_*i*_ (Figure 3B). Thus, in this model, the firing rate is assumed to be constant within each trial, but can fluctuate across trials. For all bins *t* on trial *i*, the activation (*zit*) is thus given by

(3) $$\begin{array}{l} z_{t}^{i}=b_{o}+\phi^{i}. \end{array}$$

To avoid over-fitting, we used a Gaussian-Process prior (Rasmussen and Williams, 2005) to regularize the estimates of ϕ = [ϕ_1_, ϕ_2_, ⋯ , ϕ_*K*_]. We used a Matérn kernel (Rasmussen and Williams, 2005) parametrized by a scale parameter σ*NS* and a length parameter τ*NS*; the hyper-parameters σ*NS* and τ*NS* were optimized for each cell with grid search and 10-folds cross-validation (example cross-validation landscape: Figure 3F).

#### 2.2.4. Linear Stimulus Dependence (NS-LNP)

To capture the linearly stimulus-dependent variation in spiking responses, we performed a linear convolution of an *m*-dimensional linear filter **k** with a stimulus vector **x**_*t*_ = *x*_*t*−*m*:*t*_ and added this term to the NS model (Figure 3C),

(4) $$\begin{array}{l} z_{t}^{i}=b_{o}+\phi_{i}+{\text{x}}_{t}^{⊤}\text{k}. \end{array}$$

As in the above, we used a Gaussian-Process prior to regularize the estimates of **k**. We used a Matérn kernel parametrized by a scale parameter σ*LNP* and a length parameter τ*LNP*; σ*LNP* and τ*LNP* were optimized in the same way as σ*NS* and τ*NS* for the NS model.

#### 2.2.5. Non-linear Stimulus Dependence (NS-PSTH)

Finally, to capture any other source of stimulus-dependent variability that was not accounted for by the previous two models, we included a non-parametric estimate of the firing rate in each bin *t*, ρ_*t*_ (see Figure 3D),

(5) $$\begin{array}{l} z_{t}^{i}=b_{o}+\phi_{i}+{\text{x}}_{t}^{⊤}\text{k}+\rho_{t}. \end{array}$$

Thus, in this model, we have a separate parameter for the firing rate in each bin *t*. To avoid over-fitting, we used a Gaussian-Process prior to regularize the estimates of ρ. We used an auto-regressive kernel [the covariance function of the discretized Ornstein-Uhlenbeck process (Uhlenbeck and Ornstein, 1930)] parameterized by a scale parameter σ*PSTH* and a correlation coefficient *a* (Figure 3F); σ*PSTH* and *a* were optimized in the same way as the hyper-parameters for the previous two models.

#### 2.2.6. Choice of Hyper-Parameters

The models defined above form a nested family of models, i.e., each model is a special case of the latter ones, which can be recovered by setting the additional parameters to 0. We exploited this fact when optimizing for the hyper-parameters: we first found the best σ*NS* and τ*NS*; next, keeping those fixed, we found the best σ*LNP* and τ*LNP*; finally, we optimized for σ*PSTH* and *a*.

#### 2.2.7. 10-Fold Cross Validation

We randomly generated 10-folds of trials from the data. In each fold, one or two trials were held out to be used as test data, while the remaining were used for training. We kept the folds exactly the same for all the models and all the hyper-parameter sets. We then generated a grid of values for the hyper-parameters of each model. We found the best hyper-parameters for each model in the hierarchy in order, starting from the non-stationarity model (NS). For a given hyper-parameter set from the grid, we fit the model on the training data on each fold and calculated the log-likelihood of the optimized parameters on the test data. We repeated this for each fold and averaged the calculated log-likelihood across all the folds. After calculating the average cross-validated log-likelihood(CVLL) for each hyper-parameter set, we chose the hyper-parameters corresponding to the highest average CVLL and fixed these values for the model. For the next model in the hierarchy, we only fit the parameters added to the model, while keeping the other parameters fixed at the values obtained from the previous model fit. Across all hyper-parameter sets in the new model, for each fold, the parameters from the previous model were fixed at those obtained for the best hyper-parameter set in the same fold. Additionally, after finding the best hyper-parameter set for the new model, we checked that its average CVLL was greater than the average CVLL of the best hyper-parameter set in the previous model—if this was not the case, the averaged CVLL in the new model was set to the same value as that of the previous model (however, this occurred only for 1 cell). This corresponds to choosing (0, 0) as hyper-parameter values and collapsing the new model back to the previous model (by setting the additional parameters in the new model to 0). This ensured that the new model always performed better or just as well as the previous model.

#### 2.2.8. Model Performance Measure

Following Czanner et al. (2015), we used the difference of cross validated log-likelihood between the models to quantify the improvement in model performance, and hence partitioned the response variability into different sources. The cross validated log-likelihood difference between two models is an estimate of the Kullback-Leibler distance between their predictions (Hastie, 1987). We connect the gain of each model over the model preceding it to a corresponding source of variability—for example, the log-likelihood gain of the NS model L_NS_ over the baseline model is a measure of the non-stationarity in the neural responses and the log-likelihood gain of the NS-LNP model L_LNP_ over the NS model is a measure of the linear stimulus-dependence of the neural responses.

We also calculated the gain of a saturated model L_sat_ over the most expressive model (NS-PSTH). This value allowed us to quantify how much of the total explainable log-likelihood gain was captured by our framework. The saturated model captures the average firing rate within each trial and each timebin exactly for a given dataset—this is essentially the NS-PSTH model without the linear stimulus filter, and without any regularization. The saturated model sets the upper bound for the performance that could potentially be achieved with our hierarchy of models. The log-likelihood gain of the saturated model over the NS-PSTH model, corresponds to noise or residuals that the NS-PSTH did not account for. We then calculate the signal value P_sig_ as the total log-likelihood gain of all 3 models over the baseline model (i.e., the cross-validated log-likelihood of the NS-PSTH model L_PSTH_ over the baseline model), divided by the log-likelihood gain of the saturated model over the baseline model. The noise value P_noise_ is the ratio of the log-likelihood gain of the saturated model over the NS-PSTH model, and the total log-likelihood gain of the saturated model over the baseline model, i.e.,

(6) $$\begin{array}{l} {\text{P}}_{\text{sig}}=\frac{{\text{L}}_{\text{PSTH}}-{\text{L}}_{\text{BS}}}{{\text{L}}_{\text{sat}}-{\text{L}}_{\text{BS}}} \end{array}$$

(7) $$\begin{array}{l} {\text{P}}_{\text{noise}}=1-{\text{P}}_{\text{sig}}=\frac{{\text{L}}_{\text{sat}}-{\text{L}}_{\text{PSTH}}}{{\text{L}}_{\text{sat}}-{\text{L}}_{\text{BS}}}. \end{array}$$

We also write the non-stationarity P_nonstat_, linear stimulus dependence P_lin_ and non-linear stimulus dependence P_nonlin_ as the log-likelihood gain of each model in the hierarchy over its predecessor, divided by the total log-likelihood gain of the saturated model over the baseline model.

$$\begin{array}{l} {\text{P}}_{\text{nonstat}}=\frac{{\text{L}}_{\text{NS}}-{\text{L}}_{\text{BS}}}{{\text{L}}_{\text{sat}}-{\text{L}}_{\text{BS}}},\\ \;{\text{P}}_{\text{lin}}=\frac{{\text{L}}_{\text{LNP}}-{\text{L}}_{\text{NS}}}{{\text{L}}_{\text{sat}}-{\text{L}}_{\text{BS}}},\\ \;{\text{P}}_{\text{nonlin}}=\frac{{\text{L}}_{\text{PSTH}}-{\text{L}}_{\text{LNP}}}{{\text{L}}_{\text{sat}}-{\text{L}}_{\text{BS}}}. \end{array}$$

## 3. Results

We recorded from 58 RGCs: 47 cells stimulated with 18 repeating trials of frozen and 18 trials of unique electrical stimuli in an interleaved fashion and 11 cells stimulated with 35 or 36 trials of frozen electrical stimuli. All the models were fit on the repeating trials.

We fit a hierarchy of GLMs to this data, where each model was designed to capture the non-stationarity, linear stimulus dependence and non-linearity in the spiking responses, respectively. As a measure of model performance, we compared the log-likelihood gain between different models. The different sources of variability (non-stationarity, linear stimulus dependence and non-linear stimulus dependence) are quantified by the log-likelihood gain for each model calculated from the data used to train the model. The gain of these models on data held out during training (test data) quantify the reliability of each model's predicted firing rate. As detailed in the Methods, the log-likelihood gain ranged from 0 to 1, where 0 corresponds to the cross validated log-likelihood of the baseline (BS) model, which assumed that all binned responses could be explained by the average firing rate. The maximum of 1 corresponds to the saturated model which perfectly accounts for the firing rate in each bin of every trial. Throughout this section, we report mean ± standard deviation values for the log-likelihood gain as a measure of model performance.

### 3.1. Analysis on an Example Cell

An example of the analysis pipeline can be seen in Figure 3. Figures 3B,C show the parameters captured by the NS and NS-LNP model, respectively. The progressive improvement in prediction of the firing rate is visible (Figure 3D)—the NS model does not capture any within-trial variations in firing rate; the NS-LNP model prediction is accurate only for time bins with high instantaneous firing rate and is noisy everywhere else; the NS-PSTH model does even better at capturing the peaks in the firing rate, and also at predicting periods with no spiking activity. Using the log-likelihood gain as a measure of model performance (Figure 3E), we found that the NS model (orange bar) explains a substantial part of the total log-likelihood gain both during cross validation on held out data (9.7%) and on data used to train the models (27.4%). The NS-LNP (green bar) model accounts for 3.5% of the gain on cross validation and 11.1% of the gain on training data. The NS-PSTH (red bar) model contributes the least on cross-validation with a 3.4% log-likelihood gain, but explains more of the log-likelihood gain (23%) on training data.

For this cell, using cross-validation, 83.3% of the total explainable gain is unexplained. Thus, for this cell, it seems to be the case that—beyond the strong non-stationarity, with much higher firing rates in trials 10–18—neither of the models seems to lead to substantial gains in prediction performance. Of course, it could well be the case that prediction performance is simply limited by the amount of available data used to constrain the models. If that was the case, then the prediction performance on the training-data would be substantially better. Indeed, we see here that on the training-data, up to 60% of the log-likelihood gain can be explained. Thus, from these analyses, we can e.g., conclude that between 16 and 60% percent of the gain can be captured (Figure 3E).

Hyper-parameters for regularization were set using cross validation (Figure 3F), selecting those values which lead to the best generalization performance on held-out data.

### 3.2. Quantifying Sources of Response Variability

**Figure 4:**
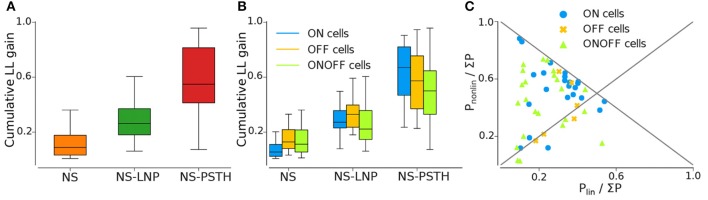
Log likelihood gain on training data for all three models. **(A)** Average log-likelihood gain across all cells. **(B)** Average log-likelihood gain partitioned by cell type. **(C)** P_nonlin_ vs. P_lin_, as a fraction of the total gain of all three models (Σ P = P_nonlin_ + P_lin_ + P_nonstat_).

**Figure 5:**
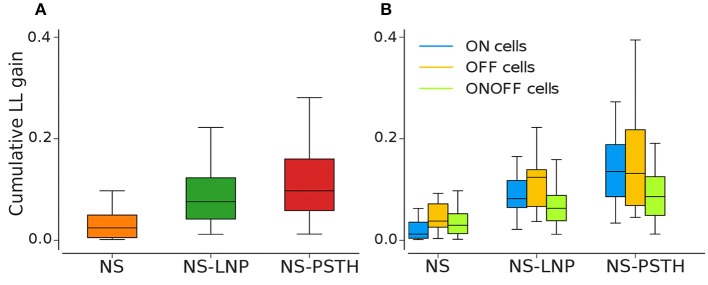
Log-likelihood gain from cross validation for all three models. **(A)** Average log likelihood gain across all cells. **(B)** Average log-likelihood gain partitioned by cell type.

Overall, our framework collectively captures 57.79 ± 3.03% of the total explainable gain on training data (Figure 4A). However, the three models together capture only 12.98 ± 4.55% of the total explainable gain when using cross validation—87.0% of the gain remains unexplained (as shown in Figure 5A and Tables 1, 2).

**Table 1:** Log-likelihood gain on training data.

	**NS**	**NS-LNP**	**NS-PSTH**	**Unexplained**
All cells	0.13	0.155	0.29	0.42
ON	0.09	0.19	0.35	0.37
OFF	0.155	0.18	0.27	0.37
ON-OFF	0.14	0.12	0.24	0.49

**Table 2:** Log-likelihood gain on test data.

	**NS**	**NS-LNP**	**NS-PSTH**	**Unexplained**
All cells	0.036	0.05	0.043	0.87
ON	0.026	0.061	0.055	0.857
OFF	0.047	0.066	0.05	0.836
ON-OFF	0.039	0.037	0.029	0.895

#### 3.2.1. Non-stationarity

Most of the cells exhibited highly non-stationary firing rates across trials (Figure 4A). This model was able to capture large scale changes in firing rate across trials—for the example cell in Figure 3, the NS model captures the abrupt increase in firing rate across trials smoothly. Overall, the NS model accounted for a mean of 12.98 ± 1.58% of the total explainable log-likelihood gain on training data (Figure 4A). In Figure 4C, NS gain over the baseline model decreases along the 45° line. Most data points lie closer to the 135° line than to the origin, implying that the NS model accounts for the least amount of log-likelihood gain in most cells. This model also captures a mean of 3.65 ± 0.49% of the total explainable gain during cross validation on average (see Figure 5A).

#### 3.2.2. Linear Stimulus Dependence

Including a linear dependence between the stimulus and the spike response, we can account for an extra 15.5 ± 1.44% of explained variability on training data and 5.00 ± 0.60% on test data.

#### 3.2.3. Non-linear Stimulus Dependence

A large fraction of explainable variability was nevertheless not yet captured by the non-stationary linear model (NS-LNP). We therefore decided to quantify the non-linear interaction between stimuli and RGC responses using the NS-PSTH model. We observed a substantial increase of explanatory power of such a model over the simpler model accounting only for linear interactions and non-stationarity (mean gain = 29.21 ± 2.44% on training data). In Figure 4C, most of the data points lie above the 45° line, implying that NS-PSTH captures a greater fraction of the gain for most cells, irrespective of cell type and that the RGCs are highly non-linear in their responses. The mean NS-PSTH model gain over NS-LNP on cross-validation data is minuscule compared to the training data (4.33 ± 0.72%), implying that the predictions of NS-PSTH do not generalize well to unobserved data.

### 3.3. Differences in Response Variability Between Different RGC Types

We also investigated the performance of our three different models grouped by cell type (Figures 4B, 5B).

ON cells were more stable than both OFF and ON-OFF cells (mean gain of the NS model was 9.23 ± 2.17%, 15.56 ± 3.82%, and 14.28 ± 2.18% on training data for ON, OFF, and ON-OFF, respectively, as shown in Tables 1, 2). ON-OFF cells were the least non-linear cells (mean NS-PSTH gain = 24.51 ± 3.32% vs. 34.79 ± 3.86%, and 27.14 ± 7.01% for ON and OFF cells, respectively). This result was indeed surprising, as we would have expected to observe a higher degree of non-linearity in ON-OFF cells for their intrinsic property of responding equally to both positive and negative stimuli. Moreover, we would have also expected to observe smaller, if at all negligible, levels of non-linearity in ON and OFF cells. On test data, the NS model had a lower log-likelihood gain for ON cells (mean gain = 2.66 ± 0.69%) compared to OFF (gain = 4.71 ± 1.12%) and ON-OFF cells (gain = 3.87 ± 0.64%)—implying that there is little information that can be gleaned about the average firing rate in a given trial from the knowledge of the firing rate in neighboring trials for ON cells. Both NS-LNP and NS-PSTH performed worst on ON-OFF cells (mean = 3.69 ± 1.01% and 2.92 ± 0.76% of total explainable gain, respectively) with cross-validation compared to ON (mean = 6.10 ± 0.84% and 5.56 ± 1.16% of total explainable gain) and OFF cells (6.65 ± 1.70% and 4.99 ± 3.12% of total explainable gain)—implying that ON-OFF cell responses are modulated by processes that are independent of the stimulus.

## 4. Discussion

Traditionally retinal prostheses have utilized large amplitude electrical pulses to elicit visual percepts. Large amplitude pulses are a natural choice as they can drive the retina with great fidelity. However, large amplitude stimulation has also been associated with indiscriminate retinal activation which can lead to an overall reduction in restored visual acuity. To tackle this, recent studies have demonstrated the ability to elicit electrically driven responses in RGCs using subthreshold electrical white-noise stimulation (Sekhar et al., 2016). Additionally, such stimulation paradigms have helped uncover a diverse set of electrical input filters that correlate well with visual cell type (Sekhar et al., 2017; Ho et al., 2018). This diversity in electrical filters, raises the possibility for cell-type specific stimulation, which would go a long way in improving the restored visual acuity. However, weaker stimulus pulses also typically run the risk of driving the retina with less fidelity. Therefore, the central goal of this study was to quantify how well subthreshold electrical stimulation is able to drive the retina, and if diversity in electrical filters comes at the expense of response fidelity. Finally in a broader scope, we believe the methods used in this study and previous studies (such as Sekhar et al., 2016, 2017; Ho et al., 2018), provide an overall framework by which one can find a set of stimulation parameters that are able to drive the retina with sufficient fidelity while still maintaining a diversity in the elicited responses.

In order to quantify how predictable the responses of retinal ganglion cells are, when stimulated electrically, we fit a hierarchy of models and tested them both on held-out data (test data) and on the training data. Performance on the training data constitutes an upper bound (i.e., an optimistic estimate) on predictability; performance on test data a lower bound (i.e., a pessimistic estimate). Data constraints from limited recording lengths can imply a substantial gap between these two bounds. While it might seem undesirable that these two bounds are not closer together, this discrepancy merely reflects the limitations of what can be concluded from limited data in the presence of variability and non-stationarity.

The model-performance measures calculated using the fits from the training data quantify the degree to which different sources of variability (i.e., non-stationarity, linearity and non-linearity) are present in a given dataset. We found that, although our hierarchy of models was able to capture some of the spiking variability, a large fraction was left unexplained by the three models, compared to previous studies using GLMs (e.g., Pillow et al., 2005). While this may seem surprising at first, these earlier studies used *visual* stimulation, which is much more effective in driving neural responses. In addition, many previous studies (Pillow et al., 2005; Truccolo et al., 2005) also used a neuron's own spike history to predict spiking activity. In separate analyses, we also found that using spiking history lead to a modest increase in model-performance. However, we did not pursue this further, since the focus of this study, was not to provide a full description of the temporal dynamics of observed variability of electrically stimulated RGCs, but rather to quantify how much of this variability could be attributed to the electrical stimulation alone, i.e., to characterize how well we can expect to selectively drive RGC activity using subthreshold electrical stimulation.

On average, we found that the NS-PSTH performs better relative to NS and NS-LNP on training data, implying that the RGCs are non-linear in their responses. On test data, the NS-LNP model yields a greater gain in explained response variability compared to NS-PSTH, implying NS-LNP generalizes better than NS-PSTH. This is not surprising since the NS-PSTH is a non-parametric model and the degree to which it generalizes will be determined only by how well it is regularized. Without a strong prior, NS-PSTH will always overfit to the training data. However, we could not identify a significant difference in the source of variability (non-stationarity, linearity or non-linearity), when comparing across the 3 cell types namely ON, OFF and ON-OFF. In conclusion, our modeling framework allows us to partition and quantify the different sources of variability in the responses of the RGCs—they are highly non-linear, but some of the response variability can be explained by a linear stimulus filter and a trial-varying offset for those cells with clear STAs.

These results could be interpreted as being consistent with a coding scheme of RGCs during prosthetic stimulation which has both linear and non-linear components. Moreover, the performance of the three models was comparable across the different ON, OFF, and ON-OFF cell types that were examined. Our study joins recent work reviewed in Rathbun et al. (2018)—in particular, a study by Maturana et al. (2018)—in using statistical modeling to understand how RGCs encode for electrical stimulation. Complementing the work on suprathreshold encoding by Maturana et al. we have examined the encoding of subthreshold stimuli. Although suprathreshold stimuli drive the retina with greater fidelity, they have the disadvantage of causing indiscriminate retinal activation. On the other hand, subthreshold stimuli have the advantage that they only weakly drive the cells, and thereby encourage multipulse integration leading to a reduction in indiscriminate single pulse activation. This in turn should encourage a cell to respond preferentially to stimuli that more closely match its intrinsic filter, therefore helping to uncover differences in the responses of different cell types to electrical stimulation (Sekhar et al., 2017; Ho et al., 2018). While these are points in favor of a subthreshold stimulus paradigm, it has the disadvantage of causing high response variability across stimulus trials, since the cells are only being weakly driven. This is evidenced by the large unexplained variance shown in Figure 3. Furthermore, to recover STAs with subthreshold electrical stimuli, we required long recording times over which non-stationarity played a larger role. Explicitly modeling a non-stationary component in the RGC response by means of the GLM framework provided an effective workaround to the major limitation that adaptation effects would have had in characterizing RGC response reliability to repeated electrical stimuli. We were hence able to quantify how well three separate sources (Figure 3E) could account for the variability observed in the RGC response, even for those RGCs which responded with low firing rates to electrical stimulation.

In summary, the subthreshold noise stimulus used here was intentionally designed as a gentle alternative to the much more aggressive suprathreshold pulses that are typically used. While this has allowed us to recover a variety of different STAs which could potentially be used for preferential stimulation, it has the undesirable consequence of intrinsically producing a much weaker stimulus-response correlation. This in turn limits our ability to model the spike train activity. It should be noted that the low predictive power of our GLMs cannot be due to deficiencies in the models, since the PSTH model by construction fully captures all stimulus-response correlations. From these results, we surmise that the particular subthreshold stimulation used here only drives RGCs very weakly. Therefore, future efforts should be geared toward finding stimulus parameters that yield better SNR (stimulus-response correlation) whilst still allowing for a diverse set of STAs by not overwhelming the retina with suprathreshold pulses. Some options to consider would be, non-Gaussian stimulus statistics or Gaussian white noise stimuli with higher means and narrower widths. However, it should be remembered that, if the mean of stimulus distribution is too high, the integrative subthreshold mechanisms revealed with this stimulus would be drowned out.

In conclusion, the methods and results of this study, add to a nascent body of work that provides a systematic and principled manner of identifying appropriate stimulus parameters for different cell classes based on factors, such as coding regime, reduction of indiscriminate activation and response reliability.

## Data Availability Statement

The datasets generated for this study are available on request to the senior authors.

## Ethics Statement

The animal study was reviewed and approved by State Authorities (Regierungspraesidium, Tuebingen), conducted under the supervision of the Tuebingen University facility for animal welfare (Einrichtung fur Tierschutz, Tier arztlichen Dienst und Labortierkunde), and NIH Guide for the Care and Use of Laboratory Animals.

## Author Contributions

SS and DR designed the experiments. SS carried out the experiments and initial data analysis. PR, GB, and JM developed the data analysis approach. PR and GB performed the analyses. SS, GB, and PR wrote the manuscript. All authors contributed to the manuscript revisions. EZ, DR, and JM supervised the research.

## Conflict of Interest

The authors declare that the research was conducted in the absence of any commercial or financial relationships that could be construed as a potential conflict of interest.
